# Hematopoietic Stem Cell Transplantation for Primary Immunodeficiencies

**DOI:** 10.3389/fped.2019.00445

**Published:** 2019-10-30

**Authors:** Andrew R. Gennery, Michael H. Albert, Mary A. Slatter, Arjan Lankester

**Affiliations:** ^1^Institute of Cellular Medicine, Newcastle University, Newcastle upon Tyne, United Kingdom; ^2^Pediatric Stem Cell Transplant Unit, Great North Children's Hospital, Newcastle upon Tyne, United Kingdom; ^3^Pediatric SCT Program, Dr. von Hauner University Children's Hospital, Ludwig-Maximilians Universität, Munich, Germany; ^4^Department of Pediatrics, Stem Cell Transplantation Program, Willem-Alexander Children's Hospital, Leiden University Medical Center, Leiden, Netherlands

**Keywords:** primary immunodeficiency, severe combined immnunodeficiency, Wiskott Aldrich syndrome, chronic granulomatous disease, conditioning

## Abstract

The field of primary immunodeficiencies has pioneered many of the advances in haematopoietic stem cell transplantation and cellular therapies over the last 50 years. The first patients to demonstrate sustained benefit and prolonged cure from the primary genetic defect following allogeneic haematopoietic stem cell transplantation were patients with primary immunodeficiencies. Although primary immunodeficiency patients began the modern era of haematopoietic stem cell transplantation, the history is nevertheless short-in answer to the question “what is the long term outcome of patients transplanted for primary immunodeficiencies?” we often have to say that we do not know. We believe that most patients who undergo haematopoietic stem cell transplantation for primary immunodeficiencies should live a normal lifespan with a fully corrected immune system. We are now beginning to understanding long term outcomes, the relationship to the underlying genetic defect, age, and pre-morbid condition of the patient at time of transplantation, stem cell source and donor, and effect of pre-transplant cytoreductive chemotherapy conditioning. The long term consequences of post-transplant complications such as graft vs. host disease, veno-occlusive disease, or immune dysregulation are also being recognized. Additionally, some genetic defects have a systemic distribution, and we are learning the natural history of these defects once the immunodeficiency has been removed.

The field of primary immunodeficiencies has pioneered the way in many of the advances in hematopoietic stem cell transplantation and cellular therapies over the last 50 years. In 1968, three patients with primary immunodeficiencies—one with Wiskott Aldrich syndrome and two with X-linked severe combined immunodeficiencies—were the first patients to demonstrate sustained benefit and prolonged cure from the primary genetic defect following allogeneic hematopoietic stem cell transplantation (1–3). The story of our specialty, whilst at the inception of hematopoietic stem cell transplantation, is thus short—in answer to the question “what is the long term outcome of patients transplanted for primary immunodeficiencies?,” we often have to say that we do not really know. We believe, in many cases, that patients who undergo hematopoietic stem cell transplantation for primary immunodeficiencies will live a normal lifespan with a fully corrected immune system. However, it is only now that we are beginning to dissect long term outcomes and the relationship to the underlying genetic defect, age and pre-morbid condition of the patient at time of transplantation, stem cell source and donor, and effect of pre-transplant cytoreductive chemotherapy conditioning (4–8). The long term consequences of post-transplant complications such as graft vs. host disease, veno-occlusive disease or immune dysregulation are also being recognized. Additionally, some genetic defects have a systemic distribution, and we are learning the natural history of these defects once the immunodeficiency has been removed.

We are hindered by dealing with small numbers of patients, rare diseases, changing protocols and transplant techniques, as well as suboptimal methods of measuring immune function and repertoire, and incomplete follow up information. Furthermore, the information we gather in our retrospective studies often pertains to historic rather than current practice (9). Importantly, we are also, for many diseases, beginning to understand the natural history of the disease without intervention with transplantation, so that, for some of the more common diseases, we are able to compare data between transplanted and non-transplanted cohorts (10–14).

Nevertheless, we are entering an era where we are beginning to understand the implications and consequences of previous treatment practice. Data that we are now gathering are important to aid our understanding of the impact of transplantation on patient survival, immune function, long term organ dysfunction/toxicity including fertility, and quality of life (*refer to chapter on Long Term Outcome and Immune Function After Hematopoietic Stem Cell Transplantation for Primary Immunodeficiency)*. It is now clear that early transplant before the onset of significant infection or organ dysfunction results in better outcomes for all immunodeficiencies (14, 15). In recent years, this knowledge and has heralded the introduction of newborn screening to identify patients with severe combined immunodeficiency before symptom onset (16) (*refer to chapter on Newborn Screening for SCID)*. Furthermore, data are emerging to suggest that best early and longer term outcomes of immune function require some degree of myeloid engraftment which reflects hematopoietic stem cell progenitor engraftment, and by implication, some form of pre-transplant conditioning (4, 6, 17, 18).

Our understanding of the underlying genetic defects has led us to realize that the degree of donor chimerism required for optimal outcome differs depending on the primary disease—a small percentage of donor myeloid chimerism in patients with RAG-deficient severe combined immunodeficiency is sufficient to restore complete T- and B-lymphocyte repertoire and function, whereas incomplete donor chimerism in patients with Wiskott-Aldrich syndrome is associated with an increased risk of autoimmunity (7). Patients with gain-of-function diseases such as STAT-1 or APDS appear to be more likely to experience recurrence of disease manifestations when complete donor chimerism is not achieved (19, 20) (*refer to chapter on Long Term Outcome and Immune Function After Hematopoietic Stem Cell Transplantation for Primary Immunodeficiency)*. Detailed information from larger cohorts of these patients, and those treated with small molecules or specific pathway inhibitors will help us to appropriately select patients for transplantation in the future (21).

An understanding of the toxicities resulting from our treatment approaches has driven the search for safer approaches to therapy, and resulted in less toxic chemotherapy conditioning regimens (22, 23), and is leading to approaches in antibody-based conditioning regimens (24, 25) (*refer to chapter on Conditioning Perspectives for Primary Immunodeficiency Stem cell Transplants*) and safer approaches to curative therapy including genetic correction of autologous cells by gene addition (26) or gene editing (*refer to chapter Autologous stem cell-based gene therapy for inherited disorders: state-of-the-art and future prospects)*. Our understanding of disease phenotype is being complicated as the genetic revolution, powered by new generation sequencing techniques and analysis of big data sets, reveals patients with new, less severe phenotypes who harbor mutations in genes previously understood to confer a severe phenotype. At the same time as transplantation becomes safer, it is also becoming clear that not everyone with a specific primary immunodeficiency will require transplantation (27). Nowhere is this dilemma sharper than in the expanding field of adolescent and young adult transplantation for primary immunodeficiency (28, 29) (*refer to chapter on HSCT in Adolescents and Young Adults with Primary Immunodeficiencies*). It should be acknowledged that most of our data pertain to treatment of infants or children, and cannot be automatically extrapolated to older patients. Recognizing who in this group of patients requires transplantation, or can be adequately managed with newly emerging immune-specific therapies is probably one of the most difficult current challenges. The need for collaboration between specialists, particularly through organizations like the Inborn Errors Working Party of the European Society for Blood and Marrow Transplantation (IEWP-EBMT), the Primary Immune Deficiency Treatment Consortium (PIDTC) of North America, the European Society for Immunodeficiencies (ESID), and Stem CEll Transplant for primary Immune Deficiencies in Europe (SCETIDE) and the harvesting of good quality data into specialist registries has never been greater.

As we celebrate 50 years of transplantation for primary immunodeficiencies, we can acknowledge the tremendous progress that we have achieved and look to a bright future for our patients fuelled by enthusiastic international professional collaborations between clinical specialists, basic scientists, patient organizations and industry. These collaborations should now focus on specific questions, identifying current knowledge (30), and formulating practical research questions for the future (31). Given the rarity of our patients, and relatively small cohorts of patients, it is only by close collaborative efforts, ideally between relevant societies such as those listed above, and careful use of combined registries data, with carefully directed questions that we will begin to gather the answers. Retrospective data harvesting has formed the foundation of our knowledge base to date, piecing together knowledge from different studies, often measuring diverse parameters. Perhaps now, building on the knowledge we have now accumulated, is the time to design prospective clinical trials for our patients, in order to accurately answer outstanding questions.

## Author Contributions

AG, MA, MS, and AL wrote and edited the manuscript.

### Conflict of Interest

The authors declare that the research was conducted in the absence of any commercial or financial relationships that could be construed as a potential conflict of interest.
